# Phase II trial of tamoxifen and goserelin in recurrent epithelial ovarian cancer

**DOI:** 10.1038/sj.bjc.6602752

**Published:** 2005-09-13

**Authors:** J Hasan, N Ton, S Mullamitha, A Clamp, A McNeilly, E Marshall, G C Jayson

**Affiliations:** 1 Cancer Research UK, Department Medical Oncology, Christie Hospital, Wilmslow Road, Withington, Manchester M20 4BX, UK; 2 MRC Human Reproductive Sciences Unit, University of Edinburgh, Chancellor's Building, Little France Crescent, Edinburgh EH16 4SB, UK; 3 Department Medical Oncology, Clatterbridge Cancer Centre, Liverpool, UK

**Keywords:** ovarian neoplasms, tamoxifen, goserelin

## Abstract

Endocrine therapy is a recognised option in the treatment of chemo-resistant ovarian cancer. We conducted a nonrandomised phase II evaluation of combination endocrine therapy with tamoxifen and goserelin in patients with advanced ovarian cancer that had recurred following chemotherapy. In total, 26 patients entered the study, of which 17 had platinum-resistant disease. The median age was 63 years and enrolled patients had received a median of three chemotherapy regimens prior to trial entry. Patients were given oral tamoxifen 20 mg twice daily on a continuous basis and subcutaneous goserelin 3.6 mg once a month until disease progression. Using the definition of endocrine response that included patients with stable disease (SD) of 6 months or greater, the overall response rate (clinical benefit rate) was 50%. This included one complete response (CR) (3.8%), two partial responses (PR) (7.7%) and 10 patients with SD (38.5%). The median progression-free interval (PFI) was 4 months (95% CI 2.4–9.6) while the median overall survival (OS) was 13.6 months (95% CI 5.5–30.6). Four patients received treatment for more than 2 years (range 1–31) and one of them is still on treatment. In none of the four patients was there any evidence of recurrent or cumulative treatment related toxicity. Treatment-limiting toxicity was not seen in any of the study population. Endocrine data demonstrated a marked suppression of luteinising hormone (LH) and follicle-stimulating hormone (FSH) to less than 4% of baseline values. No consistent correlation could be established between LH/FSH suppression and tumour response. Likewise no relationship was observed between Inhibin A/B and pro-alpha C levels and tumour response. Inhibin is unlikely to be a useful surrogate marker for response in locally advanced or metastatic ovarian cancer. Combination endocrine therapy with tamoxifen and goserelin is an active regimen in platinum-resistant ovarian cancer patients. Hormonal therapy is advantageous in its relative lack of toxicity, ease of administration and tolerability, thus making it suitable for patients with heavily pretreated disease, compromised bone marrow function and other comorbid conditions that contraindicate cytotoxic therapy as well as in patients with indolent disease.

Ovarian cancer is the leading cause of mortality from gynaecological malignancies in the western world (Greenlee *et al*, 2001). Every year 5000 women die from this disease in the UK and 25 000 in the United States. Through the administration of platinum-paclitaxel chemotherapy after expert gynaecological surgery, recent trials have demonstrated a median survival of 3 years in women with advanced ovarian cancer (McGuire *et al*, 1996; Piccart *et al*, 2000). Over 70–80% of patients present with advanced disease (stage III/IV). The majority of these patients will develop recurrent disease from which they will die and new treatment strategies are needed. In particular, the management of platinum-resistant disease poses a major problem given the limited effectiveness of nonplatinum compounds in this setting. Patients have depleted bone marrow reserves in the presence of heavily pretreated disease and poor performance status that compound the complexity of the problem.

Clinical studies of endocrine therapy in ovarian cancer have evaluated a number of agents including antioestrogens, oestrogens, progestogens, aromatase inhibitors and GnRH agonists. Tamoxifen is one of the most extensively studied compounds among these. The mechanism by which tamoxifen works in ovarian cancer is not known; however, some data suggest that the antioestrogen effect is important as other antioestrogens inhibit ER^+^ ovarian cancer cells *in vitro* (Langdon *et al*, 1994). Several studies have shown a response rate of 10–20% with oral tamoxifen in patients with platinum-resistant ovarian cancer (Slotman and Rao, 1988; Hatch *et al*, 1991; Ahlgren *et al*, 1993). Two systematic reviews have reported response rates of 13 and 9.6% respectively (Williams, 2001; Perez-Garcia and Carrasco, 2002).

Owing to the close temporal relationship between the increase in incidence of ovarian malignancies and the rise in serum gonadotropin concentrations, it has been suggested that gonadotropins are also involved in the development of ovarian tumours (Emons and Schally, 1994; Imai *et al*, 1994). However, continuous stimulation of the pituitary by chronic administration of gonadotropin-releasing hormone (GnRH) agonists like goserelin inhibits the hypothalamic–pituitary–gonadal axis by downregulation of the pituitary LH-RH receptors leading to suppression of LH and gonadal steroid production. Furthermore, treatment of patients with LH-RH, resulting in reduced gonadotropin concentrations has been associated with a 17% response rate in patients with advanced disease (Kavanagh *et al*, 1989).

Trials of LH-RH agonists in ovarian cancer have shown objective response rates ranging from 6.6 to 17.4% in various studies with response durations of the order of a few months (Lind *et al*, 1992; Sevelda *et al*, 1992; Paskeviciute *et al*, 2002). Response to goserelin was not correlated to histological grading or other tumor parameters. No significant treatment-related toxicities were seen in any of the above studies.

We were interested in the apparent paradox that tamoxifen has some antitumour activity in ovarian cancer whereas hormone replacement therapy (HRT) is deemed safe in women who have ovarian cancer (Eeles *et al*, 1991). One hypothesis to explain this is that HRT decreases the release of LH, a known mitogen for ovarian cancer; thus, the potential growth promoting effect of HRT is offset by the reduction in LH, which is usually high in postmenopausal women or in women who have undergone bilateral oophorectomy. If this is correct then the addition of goserelin to tamoxifen should be associated with greater anticancer activity. In keeping with these preclinical studies, an antiproliferative activity of tamoxifen was demonstrated in ovarian cancer cell lines. GnRH analogues have direct inhibitory effects on ovarian tumour growth that are distinct from the indirect steroid hormone-mediated effects (Emons *et al*, 1989; Connor *et al*, 1994; Imai *et al*, 1994; Grundker *et al*, 2000). These data suggest that the treatment of patients with a combined antioestrogen (tamoxifen) and an LH suppressant (goserelin) might improve the efficacy of endocrine therapy in ovarian cancer. This study was therefore set up as a phase II evaluation of combination endocrine therapy with tamoxifen and goserelin in patients with advanced ovarian cancer.

## 

### Study design

This was an open label, nonrandomised phase II study designed to determine the response rate (CR or PR or SD of greater than 6 months duration) and progression-free interval in patients with advanced ovarian cancer treated with tamoxifen and goserelin. In breast cancer the survival of patients treated with tamoxifen who had stable disease (SD) for 6 months is the same as those who attain a partial response (Howell *et al*, 1988). We therefore decided to include patients who had SD of at least 6 months in our definition of clinical response (clinical benefit rate). The study protocol was approved by the local research ethics committee. Both tamoxifen (Nolvadex®) and goserelin (Zoladex®) were supplied by Astra-Zeneca. All patients gave informed consent prior to trial entry.

### Inclusion criteria

Patients were eligible if they had histologically proven epithelial ovarian cancer that had progressed during or after completion of at least one platinum containing chemotherapy regimen, which usually included a taxane. Patients were also required to have adequate haematological reserves (Hb⩾10 g dl^−1^, WBC⩾3 × 10^9^ l^−1^, Platelets⩾100 × 10^9^ l^−1^), renal function (serum creatinine <120 *μ*M) and liver function (serum bilirubin <30 *μ*M, AST/ALT <2.5 × ULN in the absence of demonstrable liver metastases or <5 × ULN in the presence of liver metastases). Patients must have had a WHO performance status of 0–2 and bidimensionally measurable disease on X-ray, ultrasound, CT or MRI that was equal to or greater than 2 cm. Measurements must have been made within 4 weeks of trial entry. Those who had prior endocrine therapy for ovarian cancer were excluded, as were patients with significant comorbidity and/or active brain metastases. Patients were also required to stop HRT, immunotherapy or chemotherapy 4 weeks prior to trial entry.

### Patient evaluation and treatment

Pretreatment evaluation consisted of a physical examination, laboratory investigations as described above while additional samples were taken for CA125 and hormonal analysis (LH, FSH, inhibin A, inhibin B and pro-alpha C subunit of inhibin A). A pretreatment staging CT scan was performed on all patients at trial entry. Patients were reviewed at four weekly intervals for laboratory investigations and clinical, toxicity and laboratory assessment. Radiological evaluation was carried out every 3 months.

Patients were given oral tamoxifen 20 mg bd on a continuous basis and subcutaneous goserelin 3.6 mg, once a month for 6 months. Treatment was continued beyond 6 months in patients with stable or regressing disease until disease progression. No patients were excluded from response analysis or toxicity assessment. Response valuation was based on regression of bidimensionally measurable disease on CT measurement of tumours using WHO criteria.

### Hormone assays

Plasma concentrations of LH and FSH were measured by radio immunoassay previously described (Perheentupa *et al*, 2000), with assay sensitivities of 0.8 and 0.9 IU l^−1^, respectively, and within-assay variabilities of 4.6 and 5.0%, respectively. Inhibin B (Groome *et al*, 1996), inhibin A (Groome *et al*, 1994) and Pro-alpha C (Groome *et al*, 1995) concentrations were measured using two-site ELISA as described previously. The CV were <8% within plate and <10% between plates for each of these assays with sensitivities of 7.8 pg ml^−1^ for inhibin B, 2 pg ml^−1^ for inhibin A and 5 pg ml^−1^ for Pro-alpha C.

### Statistics

In total, 26 eligible patients were recruited to the study. The trial was designed to be terminated if no responses were observed in the first 14 patients. This scheme ensured that if the combination was active in 20% or more patients, the chance of erroneously rejecting the treatment after the first 14 patients was 0.044. Those who showed evidence of clinical benefit were allowed to continue treatment until disease progression, severe side effects or at patient's request to discontinue treatment.

### Baseline characteristics

In total, 26 patients entered the study. The median age of patients was 63 years (range 49–79). The median number of prior chemotherapy regimens was 3 (range 1–8). In total, 17 patients had platinum-resistant disease, defined as disease progression within 6 months of previous platinum therapy. Nine patients with platinum-sensitive disease opted for the study in preference to chemotherapy. Standard first-line therapy pre-1998 was carboplatin and cyclophosphamide. Thereafter patients were treated with carboplatin and paclitaxel unless taxanes were contraindicated on medical grounds. Over 50% of the patients had poorly differentiated tumours and multiple intra-abdominal disease sites although only a third of patients had disease that exceeded 5 cm diameter. Serous histology was the most common (50%). All patients had evidence of progressive disease at trial entry. A median of four cycles of treatment was administered per patient (range 1–31).

## RESULTS

**Figure 1:**
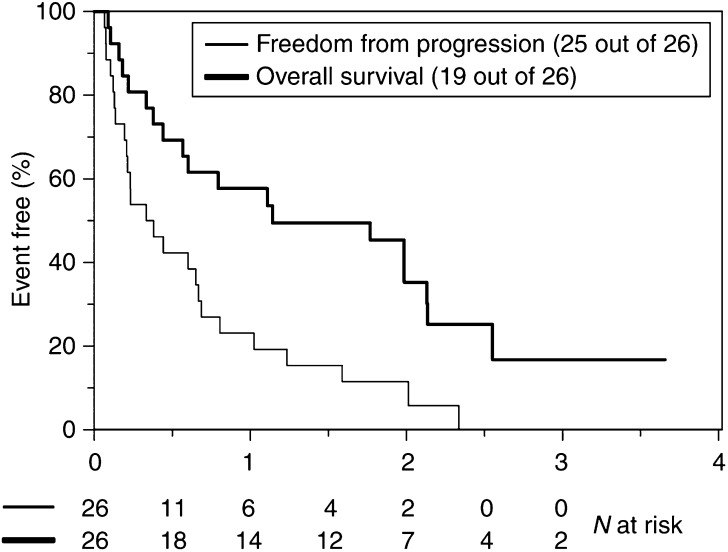
Kaplan–Meier curve for progression-free survival and overall survival with tamoxifen and goserelin. Figures below *X*-axis legend indicate patients free from progression and patients alive at six-monthly time points from the start of the treatment.

At the time of analysis, 19 of the 26 patients had died. The median follow-up on the remaining seven patients was 27.6 months (range 9.6–43.2). Using the definition of endocrine response that included patients with SD of 6 months or greater, the overall clinical benefit rate was 50%. This included one complete response (CR) (3.8%), two partial responses (PR) (7.7%) and 10 patients with SD (38.5%) that persisted for at least 6 months. The median PFI was 4 months (95% CI 2.4–9.6) (Figure 1) while the median OS was 13.6 months (95% CI 5.5–30.6) (Figure 1). There was good correlation between Ca125 and radiological response to treatment in 14 out of 15 patients on whom data was available. In only one patient, Ca125 did not match radiological response.

The treatment was well tolerated and no grade III/IV toxicities were reported. No patient was required to discontinue treatment on account of toxicity. The most common side effects were grade I–II hot flushes reported in 10 patients. Grade I abnormalities in liver enzymes were reported in two patients and weight gain in another two. A fatty liver was noted in a single patient. One patient had a pulmonary embolism that was considered to be disease-related. Four patients received treatment for more than 2 years (range 1–31). Of these three had SD and one patient had a CR. In none of the four patients was there any evidence of recurrent or cumulative treatment related toxicity.

Blood samples were taken for LH, FSH, Inhibin A and B and the pro-alpha C subunit of Inhibin and endocrine data were available on 11 patients. Of these one patient had a CR, eight had SD and two patients progressed on treatment. There was a marked suppression of LH and FSH to less than 4% of baseline values in the majority of patients. There was no significant change in serum levels of inhibin A and B post-treatment compared to pretreatment samples and all levels remained at, or below, the detection limits of the assays. Pro-alpha C levels fluctuated through the course of treatment, although an initial suppression of pro-alpha C levels was noted in all 11 patients. No consistent correlation could be established between LH/FSH suppression and tumour response. Likewise no relationship was observed between Inhibin A/B and pro-alpha C levels and tumour response.

## DISCUSSION

In the only combination therapy study to date, Hofstra *et al* (1999) evaluated the efficacy of tamoxifen 20 mg a day and goserelin 3.6 mg each month in 25 patients with chemo-resistant disease. In this case the progression free interval was 5 months (2–96) and the median overall survival (OS) was 8 months (3–96). In our study, the combination of oral tamoxifen 20 mg bd and subcutaneous goserelin 3.6 mg once a month led to one CR (3.8%), two PRs (7.7%) and 10 patients with prolonged SD (38.5%). Interestingly, the patient with a CR had platinum-resistant disease at trial entry (treatment-free interval <6 months) as did seven of 10 patients who had SD for 6 months. Data on the treatment–free interval for the two patients who experienced a partial response were unavailable. The median PFI was only 4 months, in keeping with the short response duration observed with other single-agent chemotherapy studies in this setting (Markman and Bookman, 2000). The median OS was 13.6 months with a substantial minority of patients surviving well beyond 2 years (Figure 1). Over a third of enrolled patients had SD for at least 6 months. The response rate and response duration observed in our study are significant given that the study group comprised of patients with adverse prognostic indicators and cannot be explained by selection bias or inclusion of patients with indolent disease. Indeed, the majority of patients recruited had biologically aggressive disease (>50% had platinum-resistant disease, significant tumour burden and poorly differentiated tumours. The former two being recognised adverse prognostic factors in advanced ovarian cancer). Three out of four patients who carried on with treatment for two or more years had platinum-resistant disease and multiple sites of disease. The majority of patients were heavily pretreated, having received a median of three previous chemotherapy regimens. All patients had radiological evidence of progression at trial entry.

As all patients had progressive disease at study entry, it is reasonable to conclude that combination endocrine therapy can control disease in a long-term fashion with minimal toxicity. Despite its modest efficacy, combination endocrine therapy offers an alternative option particularly in patients with heavily pretreated disease and limited bone marrow reserves and for patients with poor performance status who would not tolerate cytotoxic agents. The population recruited to this trial had received multiple courses of chemotherapy, and it would be interesting to evaluate the regimen in less chemo-resistant disease.

Endocrine analysis as expected showed a significant suppression of LH and FSH levels. However, this did not correlate with clinical response to treatment. This is in keeping with findings from recent studies that indicate the classical LH-RH receptor signal transduction pathways known to operate in the pituitary are not involved in mediating the antiproliferative effects of LH-RH analogues. Instead, these agents exert their antimitogenic effect through interference with the signal transduction of growth-factor receptors and related oncogene products associated with tyrosine-kinase activity. The mechanism of action is probably an LH-RH-induced activation of a phosphotyrosine phosphatase, counteracting the effects of receptor associated tyrosine kinase (Emons *et al*, 1998). The antiproliferative effect of GnRH analogues is also dose-dependent. Higher tissue concentrations achieved by escalating dosing regimens or alternative routes of administration may yield better responses and requires further evaluation (Emons and Schulz, 2000).

The significance of secretion of functional inhibin by epithelial ovarian cancers is not clear. Elevated serum inhibin levels have been noted in postmenopausal women with ovarian tumours (Healy *et al*, 1993) and it has been suggested that inhibin may have a role as a tumour marker, particularly when used in combination with CA125 (Robertson *et al*, 1999). Elevated serum inhibin and pro-alpha C levels have been reported in patients with GCTs and mucinous tumours of the ovary (Healy *et al*, 1993; Jobling *et al*, 1994; Boggess *et al*, 1997). Serum inhibin has never been evaluated as a marker for response to treatment in epithelial ovarian cancer although a small series has evaluated it in monitoring response to GnRH analogues in GCT (Kauppila *et al*, 1992). Available data show that inhibin assays, which detect all inhibin forms, that is, assays that detect the alpha subunit both as the free form and as an alpha-beta subunit dimer, provide the highest sensitivity and specificity for diagnosing ovarian cancer (Robertson *et al*, 2002). We therefore prospectively evaluated serum inhibin (inhibin A (alpha-betaA), inhibin B (alpha-betaB)) and pro-alpha C as potential surrogate markers of response to endocrine therapy in patients with advanced epithelial ovarian cancer. In our study, no relationship was observed between serum inhibin A/B and pro-alpha C levels and clinical response. An initial suppression of pro-alpha C levels was noted in all patients on whom data were available. The clinical significance of this remains unclear. It has been suggested that elevated serum inhibin levels in epithelial ovarian cancers are a consequence of production and secretion by the stroma rather than epithelial tumour cells (Zheng *et al*, 2000). Serum inhibin levels correlate with the extent of stroma, with stroma in mucinous tumours and sex cord tumours being more extensive than in other histological subtypes. Thus, while serum inhibin may be a useful marker for epithelial ovarian tumours confined to the ovary, it may not be appropriate in monitoring disease or response to treatment when the tumour has metastasised to other tissues.

## CONCLUSION

Combination endocrine therapy with tamoxifen and goserelin is an active regimen in platinum-resistant ovarian cancer patients. Hormonal therapy is advantageous in its relative lack of toxicity, ease of administration and tolerability, thus making it suitable for patients with heavily pretreated disease, compromised bone marrow function and other comorbid conditions that contraindicate cytotoxic therapy as well as in patients with slowly progressive disease. Prolonged survival was noted in some patients and response rates were similar to those observed with other single-agent chemotherapy, although prospective randomised studies need to be performed to confirm the superiority of this regimen.
